# NPHP proteins are binding partners of nucleoporins at the base of the primary cilium

**DOI:** 10.1371/journal.pone.0222924

**Published:** 2019-09-25

**Authors:** T. Lynne Blasius, Daisuke Takao, Kristen J. Verhey

**Affiliations:** Department of Cell and Developmental Biology, University of Michigan Medical School, Ann Arbor, Michigan, United States of America; University of Massachusetts Medical School, UNITED STATES

## Abstract

Cilia are microtubule-based organelles that protrude from the surface of eukaryotic cells to generate motility and to sense and respond to environmental cues. In order to carry out these functions, the complement of proteins in the cilium must be specific for the organelle. Regulation of protein entry into primary cilia has been shown to utilize mechanisms and components of nuclear gating, including nucleoporins of the nuclear pore complex (NPC). We show that nucleoporins also localize to the base of motile cilia on the surface of trachea epithelial cells. How nucleoporins are anchored at the cilium base has been unclear as transmembrane nucleoporins, which anchor nucleoporins at the nuclear envelope, have not been found to localize at the cilium. Here we use the directed yeast two-hybrid assay to identify direct interactions between nucleoporins and nephronophthisis proteins (NPHPs) which localize to the cilium base and contribute to cilium assembly and identity. We validate NPHP-nucleoporin interactions in mammalian cells using the knocksideways assay and demonstrate that the interactions occur at the base of the primary cilium using bimolecular fluorescence complementation. We propose that NPHP proteins anchor nucleoporins at the base of primary cilia to regulate protein entry into the organelle.

## Introduction

Cilia are microtubule-based organelles that protrude from the surface of most mammalian cells. Motile cilia function to move cells or the fluid around cells whereas primary cilia function to sense extracellular cues during development and tissue homeostasis [1–3]. Defects in the formation or function of cilia lead to numerous human diseases, termed ciliopathies [4,5], which can manifest as multi-organ syndromes [e.g. nephronophthisis (NPHP), Meckel-Gruber Syndrome (MKS), Joubert syndrome (JBTS)] with a variety of phenotypes including retinal degeneration, obesity, skeletal abnormalities, cystic organs, and situs inversus.

Cilia require a unique protein complement for their function as motile and sensory organelles. However, the cilium is not entirely enclosed by a membrane barrier but rather is open to the cytoplasm at its base. The cilium base thus acts as a gate to regulate the entry of ciliary proteins. This region is called the transition zone as this is the location where the triplet microtubules of the mother centriole transition to the doublet microtubules of the axoneme. A number of proteins identified due to their mutation in human ciliopathies localize at or near the transition zone and function in regulating the entry of ciliary proteins. Based on biochemical and genetic analyses [6–8], these proteins have been placed into four functional modules: 1) the Inversin (INVS) compartment containing NPHP2 (inversin), NPHP3, NPHP9 (NEK8), and ANKS6 (NPHP16) proteins, 2) the NPHP1/4 complex containing NPHP1, NPHP4, and NPHP8 (RPGRIP1L) proteins, 3) the NPHP5/6 complex containing NPHP5 (IQCB1) and NPHP6 (CEP290) proteins, and 4) the membrane-associated MKS complex containing MKS1, B9D1, B9D2, Tectonic1, Tectonic2, TMEM231, TMEM216, TMEM67, and CCD2A (MKS6) proteins.

Recent work has suggested that gated entry from the cytoplasm into the cilium utilizes similar molecules and mechanisms as gated entry into the nucleus [9–12]. First, a size-exclusion barrier restricts the passive diffusion of proteins larger than ~40 kDa into the nuclear compartment [13,14] and a similar size-exclusion barrier restricts protein entry into the cilium [15–18]. Second, importins and the small G protein Ran provide the selectivity and directionality, respectively, for facilitated entry of large proteins and protein complexes into the nucleus [19–21] and have also been found to regulate the ciliary localization of kinesin motors, Hedgehog signaling components, and membrane-associated proteins [22–28]. Third, nucleoporins comprise the pores in the nuclear envelope at which both the passive diffusion barrier and active transport processes regulate nucleo-cytoplasmic shuttling [29,30] and also localize to the base of primary and motile cilia and regulate entry into the cilium [16,17,31–34].

**Fig 1:**
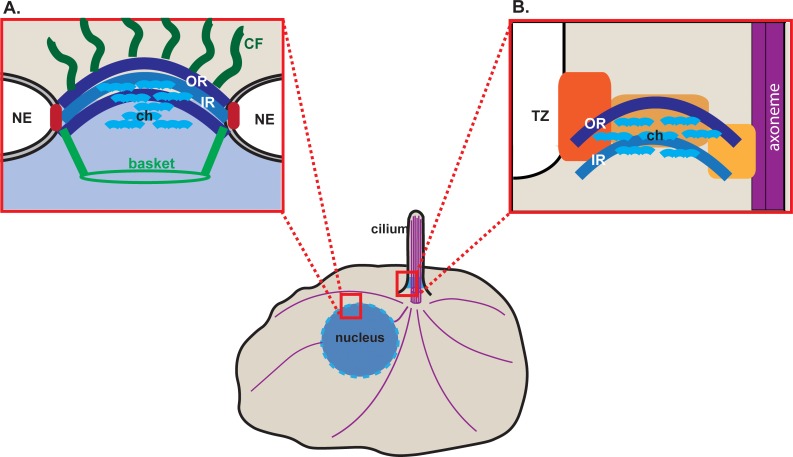
Schematic of nuclear and ciliary gating complexes. (A) At the nuclear envelope (NE), nucleoporins in NPCs are organized into subcomplexes including outer ring (OR, dark blue), inner ring (IR, medium blue), central channel (ch, light blue), cytoplasmic filaments (CF, dark green), nuclear basket (green), and transmembrane (red). (B) At the base of the primary cilium, OR, IR and channel nucleoporins localize at the transition zone (TZ) with NPHP proteins (orange). Purple lines indicate microtubules.

Each nuclear pore complex (NPC) is comprised of ~30 different nucleoporin (Nup) proteins that assemble into defined subcomplexes [29,30]. Two subcomplexes, the outer ring complex (or coat complex) and the inner ring complex (or adaptor complex), form concentric rings embedded within the nuclear envelope (Fig 1). The ring complexes surround a central channel which contains nucleoporins with intrinsically disordered regions rich in phenylalanine and glycine residues (FG Nups) that form the permeability barrier. Additional subcomplexes extend into the cytoplasm as ~50 nm long filaments or into the nucleoplasm as a basket-shaped structure.

Only a subset of the nucleoporins have been localized to the base of the cilium, in particular nucleoporins of the outer ring complex, the inner ring complex, and the central channel (Fig 1) [16,17,31–34]. However, none of the known transmembrane nucleoporins of the mammalian NPC (i.e. Pom121, gp201, Ndc1) could be localized to the base of the cilium [17], suggesting that different proteins anchor nucleoporins at the base of the cilium. Here we provide several lines of evidence that NPHP proteins bind to nucleoporins and may thus provide a scaffold for nucleoporin localization and function at the ciliary gating zone.

## Results

### Nucleoporins localize to the base of primary and motile cilia

**Fig 2:**
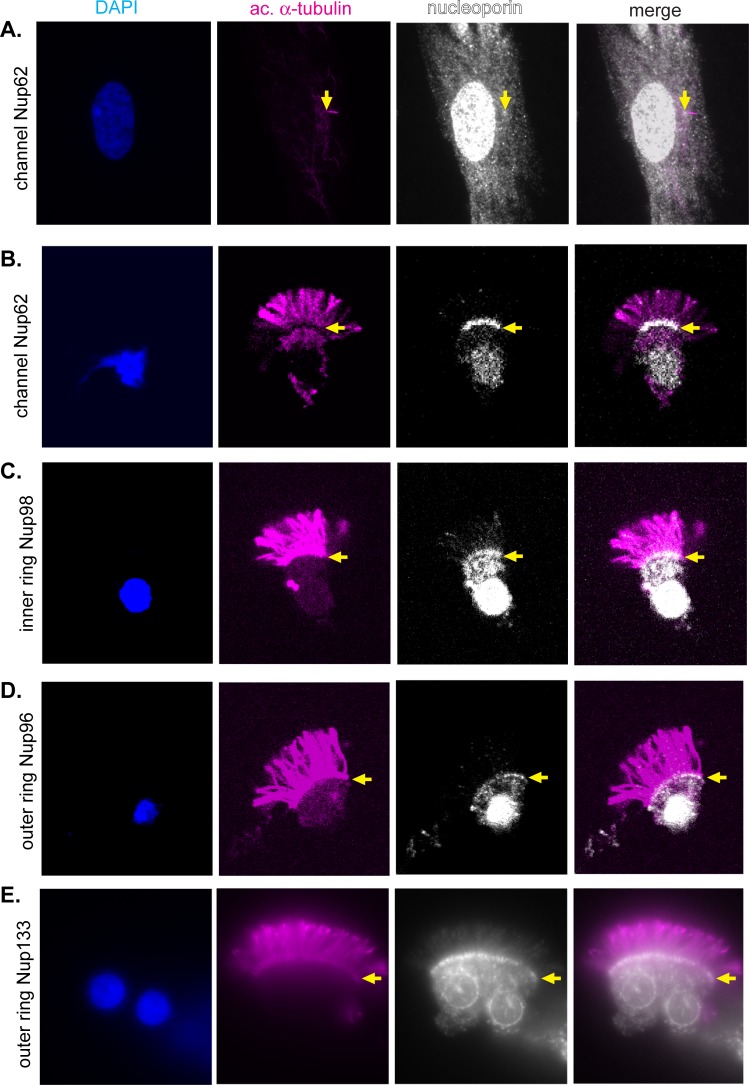
Inner ring, outer ring, and central channel nucleoporins localize to the base of motile cilia. (A) RPE-1 cells were stained with antibodies to the channel nucleoporin Nup62 (gray). (B-E) Epithelial cells isolated from rat trachea were stained with antibodies against (b) the channel nucleoporin Nup62, (C) the inner ring nucleoporin Nup98, or the outer ring nucleoporins (D) Nup96 and (E) Nup133. All cells were counter stained with antibodies to acetylated α-tubulin (magenta) to mark the ciliary axoneme and with DAPI (blue) to mark nuclei. Yellow arrows indicate the base of the cilia.

A number of nucleoporins have been localized to the base of the primary cilium using immunofluorescence including the central channel nucleoporin Nup62 (Fig 2A, [17]), the inner ring nucleoporins Nup93, Nup98, and Nup188 [16,31,32], and the outer ring nucleoporin Nup85 [16]. Whether nucleoporins also localize to the base of motile cilia has received less attention. We stained rat trachea epithelial cells with antibodies to various nucleoporins under conditions known to be favorable for immunostaining nucleoporins within the dense environment of the NPC. Antibodies to the central channel nucleoporin Nup62, the inner ring nucleoporin Nup98, and the outer ring nucleoporins Nup96 and Nup133 showed strong staining of both the nuclear envelope and the cilium base in multi-ciliated epithelial cells (Fig 2B–2E). Thus, nucleoporins likely function as ciliary gating components for both primary and motile cilia.

### Interactions between NPHP and nucleoporin proteins in a directed two-hybrid assay

A two-hybrid screen for proteins that interact with nephrocystin-4 (*C*. *elegans* homolog of NPHP4) identified npp-8 (*C*. *elegans* homolog of the inner ring nucleoporin Nup155) as an interacting protein (M. Barr, personal communication). We thus used a directed two-hybrid assay to test for interactions between mammalian NPHP and nucleoporin proteins. Full-length versions of mammalian components of the TZ modules (INVS, NPHP1/4, NPHP5/6, and MKS complexes) and nucleoporin subcomplexes (outer ring, inner ring, central channel, and cytoplasmic filaments) were fused in-frame to a DNA activating domain (AD) or a DNA binding domain (BD) and expressed in yeast of different mating types. Pairwise interactions were tested by mating strains expressing BD-fusion and AD-fusion proteins. Analysis of NPHP-NPHP and Nup-Nup pairwise matings identified known interactions (S1 Fig), confirming the validity of the assay. For example, our directed two-hybrid analysis identified previously noted interactions between NPHP1 and NPHP4 and between NPHP2 and NPHP8 [35–38] and also identified new self-interactions of NPHP2 and NPHP3 (S1A Fig). For Nup-Nup interaction analysis, many of the BD-Nup fusion proteins were transcriptionally active and thus their interactions could not be assessed in this context (S1B Fig). For the remaining nucleoporins, strong interactions were observed for the inner ring nucleoporin Nup155 with components of both the inner and outer ring complexes. Strong interactions were also observed for the outer ring nucleoporin Nup133 with components of both the inner and outer ring complexes. These results are consistent with previous two-hybrid analysis of Nup-Nup interactions as well as recent x-ray and cryo-electron microscopy structures of the mammalian NPC [39,40].

**Fig 3:**
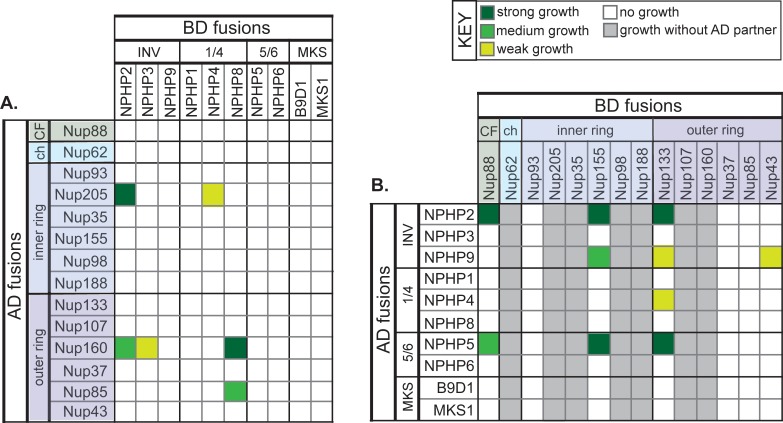
Directed yeast two-hybrid assay reveals NUP-NPHP interactions. The indicated transition zone proteins (INV, NPHP1/4, NPHP5/6, MKS complexes) and nucleoporin proteins were fused to a DNA binding domain (BD) or transcription activation domain (AD). Yeast of different mating types were mated pairwise to test for protein-protein interactions. Transcriptional activation allowing growth of diploid yeast was observed after 3 days and scored as strong growth (dark green), medium growth (medium green), or weak growth (yellow-green). Each pairwise mating was repeated at least three times. CF, cytoplasmic filament nucleoporin. ch, central channel nucleoporin.

To identify interactions between NPHP and nucleoporin proteins, matings were carried out between strains expressing BD-NPHP and AD-Nup constructs (Fig 3A) as well as between strains expressing BD-Nup and AD-NPHP constructs (Fig 3B). For the INVS module, positive interactions were observed for several members of this complex with both the outer ring and inner ring nucleoporin complexes. Specifically, NPHP2 displayed strong interactions with the inner ring nucleoporins Nup205 and Nup155, the outer ring nucleoporins Nup160 and Nup133, and the cytoplasmic filament nucleoporin Nup88 (Fig 3A and 3B). NPHP3 interacted weakly with the inner ring nucleoporin Nup160 (Fig 3A), and NPHP9 interacted with the inner ring nucleoporin Nup155 and weakly with the outer ring nucleoporins Nup133 and Nup43 (Fig 3B). For the NPHP1/4 module, positive interactions with nucleoporins were detected for two members of this complex. NPHP4 interacted weakly with the inner ring nucleoporin Nup205 and the outer ring nucleoporin Nup133 (Fig 3A and 3B), consistent with the identification of inner ring nucleoporins Nup205 and Nup93 in IP-mass spectrometry analysis of NPHP4-interacting proteins from IMCD3 cells [37]. NPHP8 interacted with the outer ring nucleoporins Nup160 and Nup85 (Fig 3A). For the NPHP5/6 complex, positive interactions were observed for NPHP5 with the inner ring nucleoporin Nup155, the outer ring nucleoporin Nup133, and the cytoplasmic filament protein Nup88 (Fig 3B). An interaction between NPHP5 and inner ring nucleoporins is consistent with previous work that identified Nup188 in IP-mass spec analysis from RPE cells [37].

### Interactions between NPHP and nucleoporin proteins in the knocksideways assay

**Fig 4:**
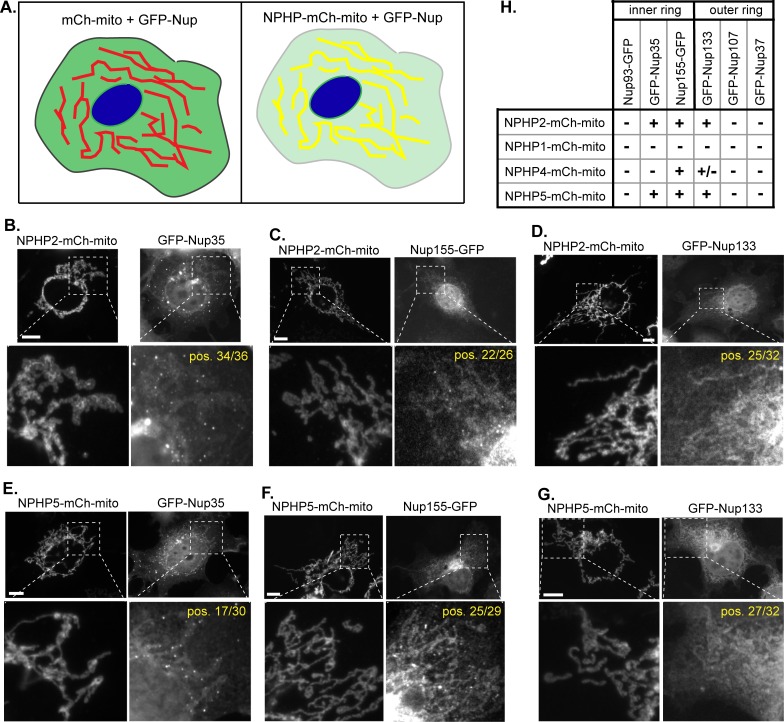
Identification of NPHP-NUP interactions by the knocksideways assay. (A) Schematic of the knocksideways assay. NPHP proteins were tagged with mCherry (mCh) and targeted to the mitochondrial surface by fusion to a mitochondrial targeting sequence (mito). The NPHP-mCh-mito bait was co-expressed with an EGFP-tagged nucleoporin (GFP-Nup). An interaction between the NPHP and nucleoporin proteins recruits the GFP-Nup to the mCh-labeled mitochondrial surface. (B-D) NPHP2-mCh-mito recruits the inner ring nucleoporins (B) GFP-Nup35 and (C) Nup155-EGFP3 and the outer ring nucleoporin (D) GFP-Nup133 to the mitochondrial surface. (E-G) NPHP5-mCh-mito recruits the inner ring nucleoporins (E) GFP-Nup35 and (F) Nup155-EGFP3 and the outer ring nucleoporin (G) GFP-Nup133 to the mitochondrial surface. Scale bars, 10 μm. Yellow text indicates the number of cells positive (pos.) for a knocksideways interaction. (H) Summary of the results. +, recruitment of GFP-Nup observed in >50% of cells; +/-, recruitment of GFP-Nup observed in 10–50% of cells; -, recruitment of GFP-Nup observed in <10% of cells.

To validate NPHP-nucleoporin interactions in a cellular context, we used the knocksideways approach (Fig 4A [41,42]). A similar approach involving the recruitment of nucleoporins to ectopic sites in mammalian cells has been utilized to analyze interactions within and between subcomplexes without disruption of the endogenous NPC architecture or function [43,44].

For our analysis, NPHP proteins were tagged with mCherry (mCh) and targeted to the mitochondrial surface by fusion with the mitochondrial targeting sequence of Omp25 (NPHP-mCh-mito fusions). We chose to focus on the INVS module protein NPHP2, the NPHP1/4 module proteins NPHP1 and NPHP4, and the NPHP5/6 module protein NPHP5 based on a) their interactions with nucleoporins in the two-hybrid assay (Fig 3) and b) their ability to localize correctly to the mitochondrial surface as mCh-mito fusion proteins (Fig 4, S2 and S3 Figs). Likewise, we chose to focus on the inner ring nucleoporins Nup93, Nup35, and Nup155 and the outer ring nucleoporins Nup133, Nup107, and Nup37 based on a) their interactions with several NPHP proteins in the two-hybrid assay (Fig 3) and b) their ability to be expressed in a soluble form in mammalian cells (Fig 4, S2 and S3 Figs).

The NPHP-mCh-mito fusion proteins were co-expressed with EGFP-tagged nucleoporin proteins in COS-7 cells. When expressed alone, the EGFP-Nup proteins localized to the nuclear envelope, as expected, but also displayed varying amounts of cytosolic, diffuse and/or annulate lamellae localization. We reasoned that co-expression with the mCh-mito control protein (Fig 4A left panel, S2 Fig) or with a non-interacting NPHP-mCh-mito protein (S3 Fig) would not alter this localization pattern whereas co-expression with an interacting NPHP-mCh-mito protein would redirect the EGFP-Nup protein to the mitochondrial surface (Fig 4A right panel).

When targeted to the mitochondrial surface, the INVS module protein NPHP2-mCh-mito was able to redistribute a portion of the inner ring nucleoporins Nup35 and Nup155 to the mitochondrion (Fig 4B, 4C and 4H), consistent with the ability of NPHP2 to interact with Nup155 in the directed two-hybrid assay (Fig 3). NPHP2-mCh-mito was also able to recruit the outer ring nucleoporin Nup133 to the mitochondrion (Fig 4D and 4H), consistent with the interaction observed in the directed two-hybrid assay (Fig 3). However, NPHP2-mCh-mito was not able to recruit the EGFP-tagged inner ring nucleoporin Nup93 nor the outer ring nucleoporins Nup107 or Nup37 to the mitochondrial surface (Fig 4H).

In a similar manner, we found that when the NPHP5/6 module protein NPHP5 was localized to the mitochondrial surface as a NPHP5-mCh-mito fusion protein, it was able to recruit the inner ring nucleoporins Nup35 and Nup155 and the outer ring nucleoporin Nup133 to the organelle (Fig 4E–4H) but was not able to recruit the inner ring nucleoporin Nup93 nor the outer ring nucleoporins Nup107 or Nup37 to the mitochondrial surface (Fig 4H). For the NPHP1/4 module, we found that NPHP1-mCh-mito was unable to recruit any of the tested nucleoporins to the mitochondrion (Fig 4H, S3A–S3C Fig) whereas NPHP4-mCh-mito was able to recruit the inner ring nucleoporin Nup155 and the outer ring nucleoporin Nup133 to the mitochondrion in some cells (Fig 4H, S3E and S3F Fig).

### Localization of NPHP-nucleoporin interactions to the base of the cilium

To determine whether interactions between NPHP and nucleoporin proteins occur at the base of the primary cilium, we utilized the bimolecular fluorescence complementation (BiFC) assay [45]. In this assay, a yellow fluorescent protein (YFP) variant is split in half, yielding N-terminal (YN) and C-terminal (YC) fragments that can be fused to proteins of interest. When expressed alone, the YN and YC fragments display little to no self-association and are non-fluorescent. However, when the YN and YC fragments are brought into close proximity via a close spatial association of their fusion partners, the fragments assemble a YFP molecule and display fluorescence. YFP fluorescence thus provides a readout of both a protein-protein interaction and the location of that interaction. A similar assay was used to verify Nup-Nup interactions within the nuclear pore complex [40] and between nucleoporins and transiting proteins at the base of the primary cilium [34].

**Fig 5:**
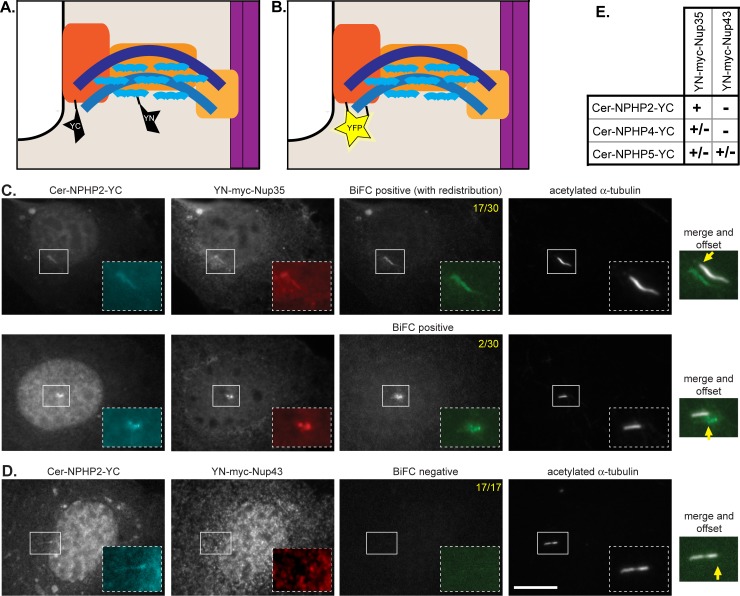
BiFC analysis reveals close association of NPHP2 and Nup35 at the base of the cilium. (A-B) Schematic of the assay. NPHP proteins (orange) were tagged with a C-terminal fragment of YFP (YC) and with Cerulean (Cer) to detect protein expression. Nucleoporin proteins (blue) were tagged with an N-terminal fragment of YFP (YN) and with a Myc-tag to detect protein expression. (A) When co-expressed in NIH 3T3 cells, the NPHP-YC and YN-NUP proteins localize to the base of the cilium but if they are not in close proximity, the YFP protein is not reconstituted (no BiFC). (B) If the NPHP-YC and YN-NUP proteins are in close proximity at the base of the cilium, then YFP is reconstituted and BiFC fluorescence is observed (right panel). (C) Co-expression of Cer-NPHP2-YC with the inner ring nucleoporin YN-myc-Nup35 results in a positive BiFC interaction. (D) Co-expression of Cer-NPHP2-YC with the outer ring nucleoporin YN-myc-Nup43 results in a positive BiFC interaction. Scale bar, 5 um. Yellow text in the top right corners indicate the number of cells displaying positive BiFC interactions across two independent experiments. Yellow arrows indicate the base of the primary cilium. (E) Summary of BiFC interactions observed for the indicated NPHP and nucleoporin proteins.

For our analysis, the NPHP proteins were tagged with Cerulean (Cer) at their N-termini to identify transfected cells and with YC at their C-termini to test for interactions with nucleoporin proteins. The nucleoporins were tagged at their N-termini with YN and with a myc tag to verify fusion protein expression. Co-expression of NPHP-YC and YN-Nup proteins that do not interact results in localization of both proteins to the base of the cilium but no YFP fluorescence (Fig 5A). In contrast, co-expression of NPHP-YC and YN-Nup proteins that localize in close spatial proximity at the base of the cilium reconstitutes YFP fluorescence at this location (Fig 5B).

For nucleoporins, the inner ring and outer ring subcomplexes are stable entities and the incorporation of an expressed, tagged subunit requires several days of protein expression and subcomplex turnover [17,40]. In addition, in our hands, localization to the nuclear envelope and cilium base is most reliable for expression of the smaller nucleoporin subunits. We thus expressed YN-tagged versions of the inner ring nucleoporin Nup35 and the outer ring nucleoporin Nup43 for this assay. For NPHP proteins, we utilized YC-tagged versions of the INVS module protein NPHP2, the NPHP1/4 module protein NPHP4, and the NPHP5/6 module protein NPHP5.

When the INVS module protein NPHP2-YC was co-expressed with the inner ring nucleoporin YN-Nup35 in NIH 3T3 cells, both proteins localized to the base of the cilium and their close proximity resulted in YFP fluorescence in 19/30 cells (BiFC signal, Fig 5C and 5E). These results suggest that NPHP2 is in close spatial proximity to the inner ring nucleoporin Nup35 at the base of the cilium. In 2 of the cells where BiFC was observed, the expressed NPHP2-YC and YN-Nup35 proteins and the BiFC signal were observed at the TZ. However, in 17 of the cells where BiFC was observed, the expressed NPHP2-YC and YN-Nup35 proteins and the BiFC signal extended from the TZ into the cilium shaft, suggesting that the irreversible nature of the BiFC interaction crosslinks the NPHP2 and Nup35 proteins and thereby alters their turnover and/or their trafficking within the cilium. When NPHP2-YC was co-expressed with the outer ring nucleoporin YN-Nup43, a BiFC signal could not be detected (0/17 cells) despite their colocalization at the base of the cilium (Fig 5D and 5E).

For the NPHP1/4 module protein NPHP4-YC, co-expression with the inner ring nucleoporin YN-Nup35 resulted in a BiFC signal at the base of the cilium in 4/35 cells whereas co-expression with the outer ring nucleoporin YN-Nup43 resulted in no BiFC signal (0/12 cells) (Fig 5E, S4A Fig). For the NPHP5/6 module protein NPHP5-YC, BiFC with the inner ring nucleoporin YN-Nup35 was observed in 8/24 cells and with the outer ring nucleoporin YN-Nup43 in 3/20 cells (Fig 5E, S4B Fig). Taken together, these results support the hypothesis that interactions between NPHP proteins and nucleoporins can serve to anchor nucleoporins at the base of the primary cilium to regulate trafficking between the cilioplasm and the cytoplasm.

## Discussion

Recent work has uncovered important roles for nucleoporins outside the NPC. In addition to their proposed role in gated entry into the cilium, nucleoporins have been implicated in regulating, for example, chromatin organization and gene expression [46,47] and in various aspects of spindle assembly, kinetochore organization, and cytokinesis during mitosis [48–51]. The complement of nucleoporins at each subcellular location, as well as their functions and spatial organization, are only beginning to be discovered and it is thought that different cellular contexts may require different nucleoporin composition [52,53]. In particular, the transmembrane nucleoporins are the least conserved across evolution and their roles have been difficult to decipher in part owing to apparent functional redundancy [30]. For example, deletion of the three known integral membrane nucleoporins in *Aspergillus nidulans* in not lethal [54] and alternative mechanisms for anchoring of outer and inner ring nucleoporins to the nuclear envelope membrane have been proposed [55–57]. Our inability to detect transmembrane nucleoporins at the base of the primary cilium [17] led us to hypothesize that cilium-specific proteins could function to anchor inner and outer ring nucleoporins at the base of the cilium. Here, we identify direct interactions of nucleoporins with NPHP proteins using a yeast two-hybrid assay and verify these interactions and co-localizations in mammalian cells. Based on these results, we hypothesize that NPHP proteins may play a functional role in the anchoring of nucleoporins at the base of the cilium.

### Nucleoporins of the outer and inner ring complexes interact with TZ proteins

**Fig 6:**
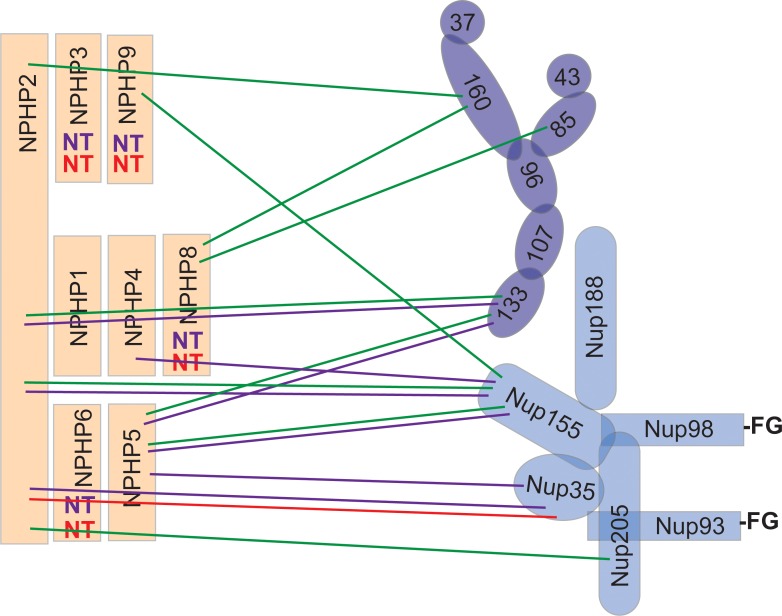
Summary of NPHP-NUP interactions. Components of the inner ring nucleoporin (medium blue), outer ring nucleoporin (dark blue), and NPHP (orange) complexes are indicated. Green lines indicate interactions detected in the directed yeast two-hybrid assay (strong and medium only). Purple lines indicate interactions detected in the knocksideways assays. The red line indicates the spatial proximity relationship observed in the BiFC assay. FG, Phenylalanine-Glycine repeats. NT, not tested.

Using a directed two-hybrid assay, we identified numerous interactions between nucleoporins of the inner and outer ring subcomplexes and NPHP proteins of the INVS, NPHP1/4, and NPHP5/6 modules (summarized in Fig 6). We then confirmed multiple interactions between nucleoporins and NPHP proteins using the knocksideways and BiFC assays in mammalian cells (summarized in Fig 6). While a negative result in any of these assays does not rule out an interaction between two proteins, these positive results provide strong support for the hypothesis that NPHP proteins interact with nucleoporins at the base of the cilium.

We identified direct interactions of the inner ring protein Nup155 with NPHP2 and NPHP9 of the INVS complex and NPHP5 of the NPHP5/6 complex. We also identified direct interactions of the inner ring protein Nup205 with NPHP2 of the INVS complex. These results are consistent with previous work where pull down/mass spec analysis identified the inner ring nucleoporins Nup188, Nup205, and Nup93 as co-precipitating with the NPHP1/4 and the NPHP5/6 complexes [37]. In the NPC, Nup155 molecules form a scaffold adjacent to the membrane of the nuclear envelope and can also bridge the inner ring and outer ring complexes whereas Nup205 is localized closer to the central channel [29,30]. It seems plausible that a similar arrangement may occur at the base of the cilium with Nup155 close to the ciliary membrane and Nup205 located near the doublet microtubules. Consistent with this hypothesis, Nup155 and other components of the inner ring subcomplex were identified as being in close proximity to tectonic and TMEM components of the membrane-associated MKS complex using a BioID-based proximity labeling approach [36].

We identified strong interactions of the outer ring protein Nup133 with NPHP2 of the INVS complex and NPHP5 of the NPHP5/6 complex. We also identified direct interactions of the outer ring protein Nup160 with NPHP2 of the INVS complex and NPHP8 of the NPHP1/4 complex. In the NPC, Nup133 and Nup160 are located adjacent to the membrane of the nuclear envelope [29,30]. At the base of the cilium, it seems plausible that these outer ring nucleoporins contact NPHP proteins close to the membrane of the primary cilium. Consistent with this prediction, nucleoporins of the outer ring subcomplex were identified in proximity mapping studies of B9D2 of the membrane-associated MKS complex [36].

### NPHP proteins in the INVS, NPHP1/4, and NPHP5/6 modules contribute to nucleoporin binding

Our directed two-hybrid analyses indicate that multiple NPHP proteins can make direct interactions with nucleoporins of the outer and inner ring complexes. A functional role for recruiting nucleoporins was confirmed for NPHP2 of the INVS module and NPHP5 of the NPHP5/6 module. Our previous work found that NPHP5 is accessible to transmembrane and signaling proteins transiting the ciliary gating zone [34], and here we report that NPHP5 interacts with nucleoporins known to localize proximal to the nuclear membrane in the NPC, suggesting that NPHP5 could form at least part of a nucleoporin anchoring complex at the outer regions of the ciliary gating zone. Furthermore, fluorescence recovery after photobleaching (FRAP) analysis demonstrated that NPHP5 displays little turnover at the base of the primary cilium [34], again consistent with a structural role in anchoring gate components.

NPHP2 is the major organizer of the INVS compartment is required for localization of NPHP3, NPHP9 and ANKS6 to this compartment [38,58–61]. NPHP2 has also been shown to co-immunoprecipitate with the NPHP1/4 module components NPHP1 and NPHP4 and is required for the proper localization of NPHP1 at the TZ [35,62,63]. We found that NPHP2 is capable of binding to a number of nucleoporins and thus may serve a more general scaffolding role at the base of the primary cilium than previously considered. NPHP2 may be membrane-associated [64,65], a finding that would place its close spatial proximity to the inner ring nucleoporin Nup35 near the membrane of the primary cilium.

The location of the INVS compartment with respect to the TZ, and its function in cilium assembly and gating, have been difficult to define. In mammalian cells, expressed GFP-NPHP2 localized to entire primary cilium in primary mouse fibroblasts and the monocilium in the mouse node [66] or at the basal body and along the length of motile cilia of mouse trachea epithelial cells [67]. In zebrafish and *C elegans*, GFP-NPHP2 localizes the proximal region of the cilium [38,65,68]. However, the GFP-tagged version of NPHP2 may not reflect the localization of the endogenous protein as the INVS compartment as marked by GFP-NPHP2 displays high variability across cells in the length of its extension along the axoneme [37]. Antibody staining for the endogenous NPHP2 protein has also produced variable results ranging from staining of the entire cilium in MDCK cells [69] to staining of the proximal region of the axoneme in polarized MDCK cells and mouse renal epithelial cells [58,61,62,64] to a spot that co-localizes with but does not extend beyond TZ markers in mouse embryo and NIH-3T3 fibroblasts [63]. Thus, there may be cell type‐specific differences in protein composition and localization of the INVS compartment.

### Nucleoporins in ciliary gating and ciliopathies

Nucleoporins of the outer ring, inner ring, cytoplasmic filament, and central channel subcomplexes have been localized to the base of the primary cilium at a location that corresponds to the TZ [16,17,31–34]. Here, we show that inner ring and central channel nucleoporins also localize as a single spot at the base of motile cilia in respiratory epithelial cells, suggesting a conserved function in regulating entry into the ciliary compartment. Localization of nucleoporins to the base of the cilium has, however, been controversial as other groups have failed to observe some of the same nucleoporins at the base of the primary cilium [15] and/or have localized nucleoporins to barrel-shaped cylinders surrounding the mother and daughter centrioles [31]. Differences in nucleoporin localization are likely due to differences in fixation and staining methods between the studies. Indeed, a recent study demonstrated that immunolocalization of a number of ciliary proteins is sensitive to the fixation and permeabilization methods utilized [70]. In our hands, immunolabeling of nucleoporins at the base of the cilium requires similar fixation methods as used for immunolabeling nucleoporins in the NPC where antibody access can be hindered by the density and biophysical properties of the NPC environment [71,72].

A role for nucleoporins in gating protein transit between the cytosol and cilium was first suggested when microinjection of function-blocking reagents decreased ciliary entry of the kinesin-2 motor KIF17 [17]. Later work used a crosslinking approach to block nucleoporin function at the base of the cilium [33]. Recent work provided definitive proof that nucleoporins at the base of the cilium determine the size-exclusion barrier as depletion of the inner ring nucleoporin Nup98 permitted the entry of proteins greater than 70 kDa into the cilium [16]. A member of the outer ring complex, Nup85, was demonstrated to be required for proper cilium localization of Nup98 [16]. Consistent with their role in determining ciliary protein content and function, mutations in nucleoporins have been found to correlate with ciliopathies in human patients. In particular, a duplication of the inner ring component Nup188 was found in a patient with heterotaxy in which altered left-right patterning of the internal organs can lead to a severe form of congenital heart disease [73]. Further work showed that depletion of the inner ring components Nup188 and Nup93 resulted in an inability to generate primary cilia in cultured mammalian cells and motile cilia in the epidermis of *Xenopus* embryos [73]. Ciliary functions of nucleoporins are thus an important consideration when linking patient mutations in nucleoporins to the underlying biology of the cell.

## Materials and methods

### Plasmids

Bait and prey constructs for the directed yeast two-hybrid assay were made by subcloning nucleoporin and NPHP genes into the pGBKT7 and pACT2 vectors, respectively, using convenient restriction enzyme sites or by amplification by PCR followed by subcloning using convenient restriction sites. Nucleoporin sequences used for subcloning were obtained from the following sources: EGFP-HsNup35, EGFP-HsNup37, EGFP-HsNup43, MmNup62-EGFP3, HsNup93-EGFP3, and EGFP-HsNup98 (EUROSCARF); HsNup155 (Mammalian Gene Collection MGC:33787 IMAGE: 5295664), MmNup188 (MGC:106061 IMAGE: 4916413), HsNup205 (MGC:168237 IMAGE:9020614), and HsNup88 (MGC:8530 IMAGE:2822595) from Dharmacon; pSV2-Lac1-CFP-MmNup85 and EGFP-HsNup160 (gifts of Dr. Douglass Forbes); EGFP-HsNup107 and EGFP-HsNup133 (gifts of Dr. Kyle Roux). NPHP sequences used for subcloning were obtained from the following sources: pENTR-HsNPHP3, -MmNPHP5, and -HsNPHP8 (gifts of Dr. Peter Jackson); pENTR-NPHP1, pENTR-NPHP2, pENTR-NPHP4 (gifts of Dr. Friedhelm Hildebrandt); pGLAP5-B9D1 (gift of Dr. Jeremy Reiter); pEGFP-mCep290/NPHP6 (gift of Dr. Joseph Gleeson, Addgene plasmid #27379 RRID:Addgene_27379); pENTR-MKS1 and pENTER-Nek8/NPHP9 (gifts of Drs. Tao Xu and Jean-Francois Rual).

For the knock sideways assays, the sequence encoding amino acids 170–206 of rat Omp25 (GenBank: AF107295.1 [74]) was synthesized (Life Technologies) and subcloned into the mCherry-C1 vector (Clontech). To generate NPHP2-mCherry-Omp25, NPHP4-mCherry Omp25, and NPHP5-mCherry-Omp25, the coding sequences of NPHP2, NPHP4 or NPHP5 were inserted using convenient restriction enzyme sites.

For the bimolecular fluorescence complementation (BiFC) assays, we found that an N-terminal fragment (aa 1–172) of the YFP variant mCitrine expressed with the C-terminal fragment (aa 155–238) of the YFP variant Venus resulted in low self-assembly (and low background fluorescence) but high BiFC fluorescence [34,75]. NPHP proteins were tagged with the fluorescent protein Cerulean (Cer) to assess protein expression and localization and with the YFP C-terminal (YC, C-terminal half of Venus) fragment. Cer-NPHP4-YC and Cer-NPHP5-YC were described previously [34] and Cer-NPHP1-YC and Cer-NPHP2-YC were constructed in a similar manner. Nucleoporin proteins were tagged with the YFP N-terminal (YN, N-terminal half of mCtrine) fragment by synthesizing a DNA sequence encoding YN followed by a flexible linker (aa sequence GGSG) and a myc tag (EQKLISEEDL) (Life Technologies) that was used to replace the EGFP sequence in the plasmids EGFP-HsNup35, EGFP-HsNup37, and EGFP-HsNup43.

### Directed yeast two-hybrid assay

Yeast two-hybrid bait and prey plasmids were transformed into yeast strains AH109 and Y187, respectively, and protein-protein interactions were assessed by yeast mating. Briefly, yeast strain AH109 expressing pGBKT7-Nup, pGBKT7-NPHP, or empty vector constructs was mated to yeast strain Y187 expressing pACT2-Nup, pACT2-NPHP, or empty vector constructs in 96 well plates. Diploid yeast were subsequently plated on double (-leu,-trp) and triple (-leu,-trp,-his) dropout plates and screened for growth two days later. Successful mating was indicated by growth on -leu,-trp drop out plates. Positive Nup-Nup, NPHP-NPHP, and Nup-NPHP interactions were evidenced by growth on -leu,-trp,-his plates. Strong interactions gave rise to yeast colonies within two days of plating while weaker interactions appeared after three days.

### Cell culture

COS-7 cells (purchased from ATCC: CRL-1651) and NIH-3T3 cells (purchased from ATCC: CRL-1658) were cultured in DMEM (Gibco) supplemented with 10% Fetal Clone III (Hyclone) and 1% GlutaMAX (Gibco) and transiently transfected with Trans-IT LT1 (Mirus). NIH-3T3 cells were transferred to serum-free medium immediately prior to transfection to accumulate cells in G1 for visualization of the primary cilium.

### Antibodies and microscopy

Antibodies were purchased against K40-acetylated α-tubulin (mouse monoclonal 6-11B-1, Sigma T7451; 1:10,000), myc epitope tag (rabbit polyclonal Sigma C3956; 1:500), Nup62 (mouse monoclonal BD Biosciences 610497; 1:250), Nup96 (rabbit polyclonal NOVUS NB100-93325; 1:250), and Nup133 (rabbit polyclonal Bethyl A302-385A; 1:750). Rabbit polyclonal antibodies against Nup98 were a gift from Dr. Richard Wozniak (1:2500). Secondary antibodies were from Jackson ImmunoResearch (1:500).

The trachea was dissected from adult rats and epithelial cells were dissociated with a toothpick, spun onto coverslips, and immediately fixed with 3.7% formaldehyde in PBS for 10 minutes and quenched with 50 mM NH4Cl/PBS for 5 min. The cells were blocked, washed, and incubated with antibodies in IF buffer (0.1% Triton X-100, 0.02% SDS, 10 mg/ml BSA in PBS) and mounted using Prolong Gold (Invitrogen). Confocal imaging was performed on Leica SP5X and Olympus Fluoview 500 confocal microscopes with a 60 x 1.40 numerical aperture (N.A.) objective. All experimental procedures were approved by the University of Michigan Committee on the Use and Care of Animals (#PRO00008901) and performed in accordance with the recommendations in the Guide for the Care and Use of Laboratory Animals of the National Institutes of Health.

COS-7 cells were transfected with NPHP-mCherry-Mito and EGFP-NUP plasmids and fixed 24 hours post-transfection with 3.7% formaldehyde in PBS, quenched with 50 mM NH_4_Cl/PBS, then mounted using Prolong Gold (Invitrogen) and imaged. NIH-3T3 cells were transfected with Cer-NPHP-YC and YN-myc-Nup plasmids and then fixed 48 hours post-transfection with 3.7% formaldehyde/PBS, quenched with 50 mM NH_4_Cl/PBS, and then blocked, washed, and incubated with antibodies in IF buffer. All antibody incubations were for 1 hour, followed by three washes of IF buffer. Fluorescence images were obtained using an inverted epifluorescence microscope (Nikon TE2000-E) with 60x oil immersion objective (N.A. 1.4) and a Photometrics Cool Snap HQ camera.

## Supporting information

S1 Fig Directed yeast two-hybrid analysis of NPHP-NPHP and NUP-NUP interactions. Yeast of different mating types expressing proteins fused to a DNA binding domain (BD) or transcription activation domain (AD) were mated pairwise. Transcriptional activation allowing growth of diploid yeast was observed after 3 days. (A) NPHP-NPHP interaction analysis. (B) NUP-NUP interaction analysis.
(EPS)

S2 Fig Control mCh-mito construct for knocksideways assay. The mCh-mito construct localizes to the mitochondrial surface but fails to recruit the inner ring nucleoporins (A) GFP-Nup35 and (B) Nup155-EGFP3 or the outer ring nucleoporin (C) GFP-Nup133 to the mitochondrial surface.
(EPS)

S3 Fig Analysis of NPHP1 and NPHP4 in the knocksideways assay. (A-C) NPHP1-mCh-mito localizes to the mitochondrial surface but fails to recruit the inner ring nucleoporins (A) GFP-Nup35 and (C) Nup155-EGFP3 or the outer ring nucleoporin (C) GFP-Nup133 to the mitochondrial surface. (D-F) NPHP4-mCh-mito localizes to the mitochondrial surface but fails to recruit the inner ring nucleoporin (D) GFP-Nup35 and the outer ring nucleoporin (F) GFP-Nup133 but does recruit the inner ring nucleoporin (E) Nup155-GFP to the mitochondrial surface. Scale bars, 10 μm. Yellow text indicates the number of cells positive (pos.) or negative (neg.) for a knocksideways interaction.
(EPS)

S4 Fig BiFC analysis of NPHP4 and NPHP5 with nucleoporins. (A) Co-expression of Cer-NPHP4-YC with the inner ring nucleoporin YN-myc-Nup35 results in a positive BiFC interaction in 4/30 cells. (B) Co-expression of Cer-NPHP5-YC with the inner ring nucleoporin YN-myc-Nup35 results in a positive BiFC interaction in 8/24 cells. Scale bar, 5 um. Yellow text in the top right corners indicate the number of cells scoring negative or positive for a BiFC interaction across two independent experiments. Yellow arrows indicate the base of the primary cilium.
(EPS)
